# Real-time crop row detection using computer vision- application in agricultural robots

**DOI:** 10.3389/frai.2024.1435686

**Published:** 2024-10-30

**Authors:** Md. Nazmuzzaman Khan, Adibuzzaman Rahi, Veera P. Rajendran, Mohammad Al Hasan, Sohel Anwar

**Affiliations:** ^1^Lead Research Scientist (Kroger), 84.51°, Cincinnati, OH, United States; ^2^Mechatronics and Autonomous Research Lab, Purdue University, Mechanical Engineering, Indianapolis, IN, United States; ^3^Engineering, Research & Development, Equipment Technologies Inc., Mooresville, IN, United States; ^4^Department of Computer Science, Luddy School of Informatics, Computing, and Engineering, Indiana University, Indianapolis, IN, United States

**Keywords:** crop row detection, precision farming, agricultural robot, unsupervised learning, real-time application

## Abstract

The goal of achieving autonomous navigation for agricultural robots poses significant challenges, mostly arising from the substantial natural variations in crop row images as a result of weather conditions and the growth stages of crops. The processing of the detection algorithm also must be significantly low for real-time applications. In order to address the aforementioned requirements, we propose a crop row detection algorithm that has the following features: Firstly, a projective transformation is applied to transform the camera view and a color-based segmentation is employed to distinguish crop and weed from the background. Secondly, a clustering algorithm is used to differentiate between the crop and weed pixels. Lastly, a robust line-fitting approach is implemented to detect crop rows. The proposed algorithm is evaluated throughout a diverse range of scenarios, and its efficacy is assessed in comparison to four distinct existing solutions. The algorithm achieves an overall intersection over union (IOU) of 0.73 and exhibits robustness in challenging scenarios with high weed growth. The experiments conducted on real-time video featuring challenging scenarios show that our proposed algorithm exhibits a detection accuracy of over 90% and is a viable option for real-time implementation. With the high accuracy and low inference time, the proposed methodology offers a viable solution for autonomous navigation of agricultural robots in a crop field without damaging the crop and thus can serve as a foundation for future research.

## 1 Introduction

Autonomous navigation in agriculture has several advantages, such as reduced operator fatigue, improved profit and efficiency, and enhanced operation safety. To support autonomous navigation, a crucial task is to develop the capability of computer-vision based detection of crop rows from image/video data. To address this, several methodologies have already been proposed (Basso and de Freitas, 2019; Hough, 1962; Zhang et al., 2018; Sainz-Costa et al., 2011); however, our analysis of these existing methodologies reveals that their detection quality deteriorates significantly when the crop row image is complex. Specifically, the complexity arises due to two reasons: first, discontinuity in a crop row due to missing crops within a segment of a crop row, and second, considerable weed growth in the area between the rows of crops. When a frame of a video feed contains both of the aforementioned issues simultaneously, the distinction between crop rows and weeds becomes highly challenging, and the majority of the existing approaches are unable to achieve satisfactory performance in addressing the task of crop row detection.

The prerequisites of a viable crop row detection method that effectively operates in real-life deployment are as follows: (1) It demonstrates the ability to detect crop rows even in the presence of significant weed pressure; (2) It is suitable for implementation in various types of crop fields; (3) It is capable of detecting crop rows at different stages of crop growth; (4) It can accurately identify both straight and moderately curved crop rows; and finally, (5) The processing time required for detection on an off-the-shelf computer meets the real-time requirements. In this work, we propose a Clustering Algorithm based RObust LIne Fitting (CAROLIF) crop row detection method which satisfies all the above requirements.

Different studies have been conducted for crop-row detection using different methods. Basso and de Freitas (2019) used a filtered Hough transform (Hough, 1962) method and achieved around 30 frames per second (FPS) for images with 320 × 240 resolution on an RPi-3B embedded system. Although the study presented comprehensive findings about the algorithm's performance across different image sizes and frame rates (the maximum speed used for good detection: 2 m/s), no detailed calculation is shown about the effect of weed pressure and missing crop on row detection accuracy. Zhang et al. (2018) proposed a double thresholding (combining Otsu's method with particle swarm optimization) on segmented image with linear regression for line fitting. The authors said that their system is capable of differentiating between weed and crop rows through the use of double thresholding. However, they did not provide details regarding the performance of their method in conditions of heavy weed pressure. Also, least squares fitting is highly affected by the presence of weed in the image. Sainz-Costa et al. (2011) developed a strategy based on the analysis of video sequences to detect crop rows. They used the lower half part of the image for detection. The image processing experienced five steps: segmentation, morphological opening, horizontal strips dividing, vertical averaging, row centers extracting and crop rows finding. The above approach can work well under low weed pressure. Chen et al. (2021) constructed a method to use UAV images to detect crop rows. The authors experimented on cotton and wheat fields to test their algorithm and found Crop row detection accuracy (CRDA) of 0.99–1.00 for cotton fields and 0.66–0.82 for wheat fields. But the computations were done on orthomosaic images only and neither curved crop rows nor weed presence was considered. Vidović et al. (2016) applied the vanishing point principle to determine that parallel crop rows (straight and curved) are preserving the inter-crop row distances (regular patterns), which allows the application of a matching technique to combine image evidence and prior knowledge about the geometric structure. Optimization, based on dynamic programming, was applied for straight and curved crop row detection for different row crop. However, an image with resolution of 640 × 480 needed around 5 s of processing time. Li et al. (2022) followed a similar approach where they used vanishing point and line-scanning method to detect hybrid rice rows at pollination stage to get an Intersection over Union (IoU) of 0.832 and an *F*_1_ score of 92.23%. But the authors did not consider weed presence in their domain of study. Garćıa-Santillán et al. (2018) proposed a method consisting of image processing by Otsu thresholding, Hough Transform, Morphological operations and regression analysis to detect crop rows. The method performs well under different operating conditions, but required 7 s to process one image, which makes it unsuitable for real-world implementation. Rabab et al. (2020) used projective transformation and segmentation with connected component analysis to connect binary objects which are supposed to be crop rows. They used a distance threshold value to determine which binary objects belong to same crop row. Their algorithm shows better accuracy than other state-of-the-art methods, but lacks viability of real-time application due to its long processing time (0.7 s). Also how the algorithm may perform in a dynamic environment with challenging situations from continuous video is not discussed. Gai et al. (2021) developed an under-canopy navigation system using Time-of-Flight (ToF) camera to map and detect parallel crop rows. They used a robotic ground vehicle specifically designed for an agronomic row spacing of 0.76 m. Yang et al. (2022) proposed a method based on color transformation, determination of multiple Region of Interest (ROI), extraction of feature points and line fitting using least-squares. The authors tested the algorithm on a maize field during the tasseling stage and achieved an accuracy of 98.6%. But a considerably high computation time of 0.3 s and reduction of accuracy for different crop-row spacing makes the algorithm less suitable for generalized application. Winterhalter et al. (2018) used pattern hough transform where they determined a pattern that is best supported by all data in contrast to (incrementally) extracting single lines. Their method showed good results for tiny sugar beet plants. But for plants with bigger leaves like Canola, their success rate dropped drastically. Also how the presence of weed can affect the algorithm is not discussed. Ota et al. (2022) used RGB-D images taken from a cabbage field to remove plant area from depth image and fitting the wave surface to detect crop rows. Although their proposed method considers weed pressure, the study did not mention any computation time or real-time implementation. Pang et al. (2020) proposed an Region-based Convolutional Neural Network (RCNN) algorithm and used UAV to capture images to detect rows on a maize field and achieved a maximum accuracy of 95.8%, but the study did not consider the scenarios where weed can be present on the field. Table 1 provides a comparative overview of the discussed methods. Overall, more research is needed to develop an algorithm which can show robust performance in difficult scenarios (shadow, high weed pressure, missing crop row) and viable for real-time application (low inference time).

**Table 1:** Comparison of crop row detection methods.

**References**	**Method**	**Crop types**	**Performance metrics**	**Considerations**
Sainz-Costa et al. (2011)	Video sequence analysis	Not specified	Avg dist: 20 px, Std dev: 28.5 px	Less effective under high weed pressure
Vidović et al. (2016)	Vanishing point with dynamic programming	Various crops	0.2 FPS (640x480)	Processing time unsuitable for real-time use
Garćıa-Santillán et al. (2018)	Otsu thresholding, Hough Transform, morphological ops, regression	Maize	0.143 FPS	Slow processing, not for real-time use
Winterhalter et al. (2018)	Pattern Hough Transform	Canola, Corn, Leek, Sugar Beet	11.2–24.4 FPS (varies by crop)	Lower performance for plants with larger leaves; weed impact not discussed
Zhang et al. (2018)	Double thresholding with linear regression	Maize	CRDA: 0.5°	Affected by weed presence; no details for heavy weed pressure
Basso and de Freitas (2019)	Filtered Hough Transform	Maize	30 FPS (320 × 240)	No details on weed pressure or missing crop impact
Pang et al. (2020)	RCNN with UAV images	Maize	Accuracy: 95.8%	Weed presence not considered
Rabab et al. (2020)	Projective transformation and segmentation	Various crops	1.43 FPS	Long processing time; real-time suitability unclear
Chen et al. (2021)	UAV images with least-square method	Cotton, Wheat	CRDA: 0.99–1.00 (cotton), 0.66–0.82 (wheat)	Based on orthomosaic images; no consideration for curved rows or weeds
Gai et al. (2021)	ToF camera for crop row mapping	Corn, Sorghum	MAE: 3.4 cm (Corn), 3.6 cm (Sorghum)	Optimized for specific row spacing (0.76 m)
Li et al. (2022)	Vanishing point and line-scanning	Hybrid rice	IoU: 0.832, *F*_1_: 92.23%	No weed consideration
Ota et al. (2022)	RGB-D with wave surface fitting	Cabbage	CRDA: 1.15° (crop), 1.72° (weed); *F*_2_: 0.919 (crop), *F*_0.5_: 0.999 (weed)	No computation time or real-time details
Yang et al. (2022)	Color transformation, ROI, line fitting	Maize	Accuracy: 98.6%, 3.33 FPS	High computation time; lower accuracy for varying row spacing

**Figure 1:**
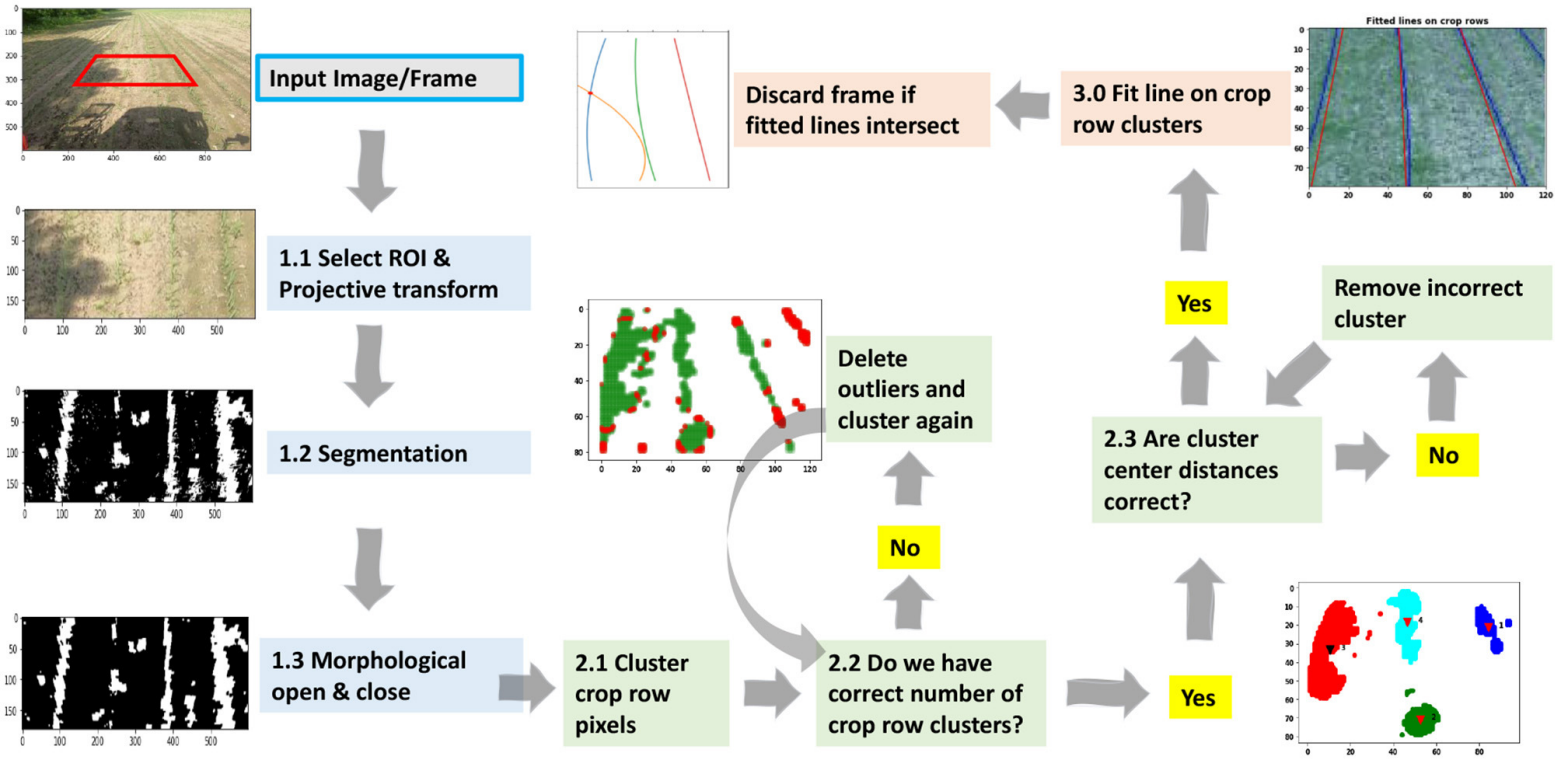
Steps of the proposed CAROLIF algorithm with images.

The basic concept underlying our proposed methodology is the utilization of an effective clustering technique to distinguish between green weed pixels, which are considered noise points, and green crop row pixels, which are identified as data clusters. The primary aim of this study is to accurately classify each crop row as a separate cluster in order to facilitate the fitting of a regression line inside each cluster. This regression line will then be returned as the identified crop row. In the first step, the input image undergoes a series of operations. Firstly, the input image is cropped, transformed to an image plane vertical to the crop rows, segmented to separate the desired crop from the background, and cleared from small noise or weed segments. At this point, the image has two channels, black and white pixels. The black pixels represent the background, while the white pixels correspond to crop and weed. Now the goal is to differentiate weed pixels from crop pixels with the purpose of afterwards fitting lines to the crop rows. A clustering algorithm is implemented to cluster the pixels of each crop row and distinguish weed pixels from the resulting clusters. The presence of weed pixels within the crop row cluster has the potential to impede the precision of the line-fitting algorithm. As a result, a robust line-fitting algorithm is employed to fit a line onto each cluster of crop rows, thereby eliminating the effects of outlier points (weed or noise). Finally, the lines that have been fitted are plotted on the image and returned as detected crop rows. All the steps of the proposed algorithm are visually presented in Figure 1.

## 2 Data collection

To evaluate our algorithm and compare it with benchmark methods, we used both an image dataset and video feeds.

### 2.1 Image dataset

**Figure 2:**
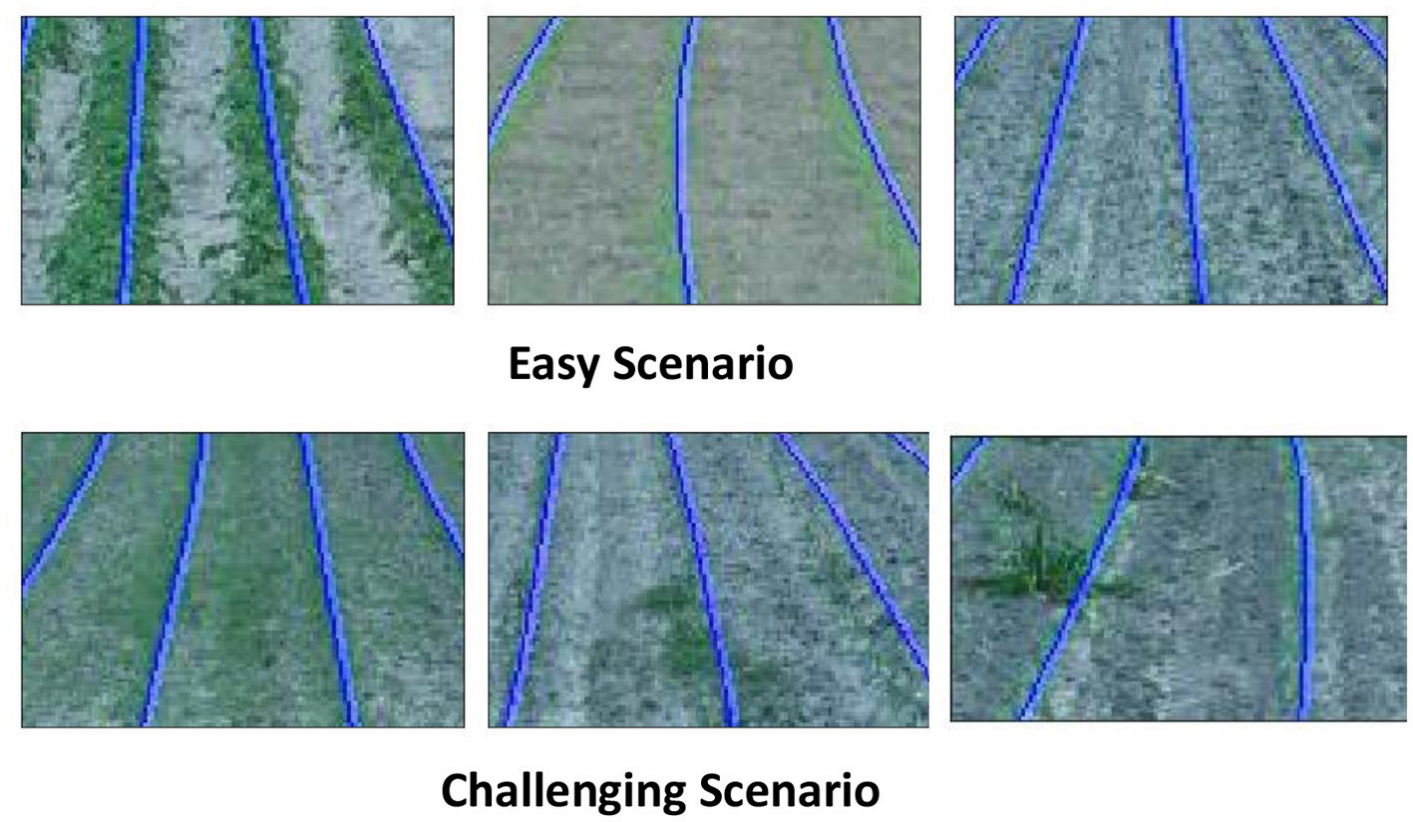
Example cases for easy and challenging scenario.

We selected thirty distinct images from the CRBD dataset to test the effectiveness of the proposed method. Two specific scenarios were identified for analysis. "Easy scenarios" are characterized by well-spaced crop rows with minimal or no weed growth, where the rows are either straight or have moderate curvature. "Challenging scenarios" involve sporadic or absent crop growth in certain rows, a significant presence of weeds, interconnection of crop rows due to weed overgrowth, and pronounced curvature in the crop rows. Figure 2 illustrates examples of both easy and challenging scenarios.

### 2.2 Video dataset

To assess the algorithm's efficacy in a real-time setting, we utilized a video dataset. The footage was captured with a GoPro Hero8 camera, which offers 4K resolution and records video at 60 frames per second. Detailed specifications of the camera are provided in Table 2. The camera was mounted at the front of a tractor, angled at ~35 degrees, while the tractor moved at a speed of around 10 miles per hour. The video begins with the tractor operating in an environment characterized by straight crop rows and optimal detection conditions. About one minute into the recording, the tractor makes a slight left turn. The final 30 s of the video showcase a challenging environment, where crop row detection is hindered by shadows, inconsistent crop growth, and a substantial presence of weeds. The footage was recorded in May 2020 in a cornfield located in Indianapolis, Indiana, under clear, sunny weather conditions.

**Table 2:** Specification of video camera.

**Parameter**	**Value**
Pixels	12 Megapixel
Aspect ratio	16:9
Vertical field of view	69.5°
Horizontal field of view	118.2°
Video capture rate	4K@60 FPS

## 3 Methodology

### 3.1 Development of the algorithm

Below is described the process how the proposed algorithm works.

#### 3.1.1 Step 1: Pre-processing

First a region of interest (ROI) is selected with a minimum of three crop rows. All the algorithmic methods are applied within this ROI. There are three benefits to selecting this ROI. First, green pixels near the horizon are congested and are challenging to separate, resulting in increased false detection. By choosing this ROI, the requirement to process the green pixels is eliminated. Secondly, this ROI is approximately one-fourth of the dimensions of the original image. Restricting operations to this section results in a reduction in computational cost and processing time. And third, curved rows within this limited ROI are reasonably straight. Projective transformation is employed to convert the crop rows converging at infinity into parallel lines. Consequently, fitting straight lines to these crop rows yields satisfactory outcomes.

##### 3.1.1.1 Projective transformation

The term “projective transformation” refers to the mapping that associates a set of points from one image plane with a corresponding set of points on another image plane. Planar homography refers to the projective mapping from one plane to another. Hartley and Zisserman (2003) provide a definition of projective transformation as follows: “A planar projective transformation is a linear transformation on homogeneous 3-vectors represented by a non-singular 3-by-3 matrix.”


(1) $$\begin{array}{l} \left(\begin{array}{c} x_{1}^{\prime }\\ x_{2}^{\prime }\\ x_{3}^{\prime } \end{array}\right)=\left[\begin{array}{ccc} h_{11} & h_{12} & h_{13}\\ h_{21} & h_{22} & h_{23}\\ h_{31} & h_{32} & h_{33} \end{array}\right]\left(\begin{array}{c} x_{1}\\ x_{2}\\ x_{3} \end{array}\right) \end{array}$$

**Figure 3:**
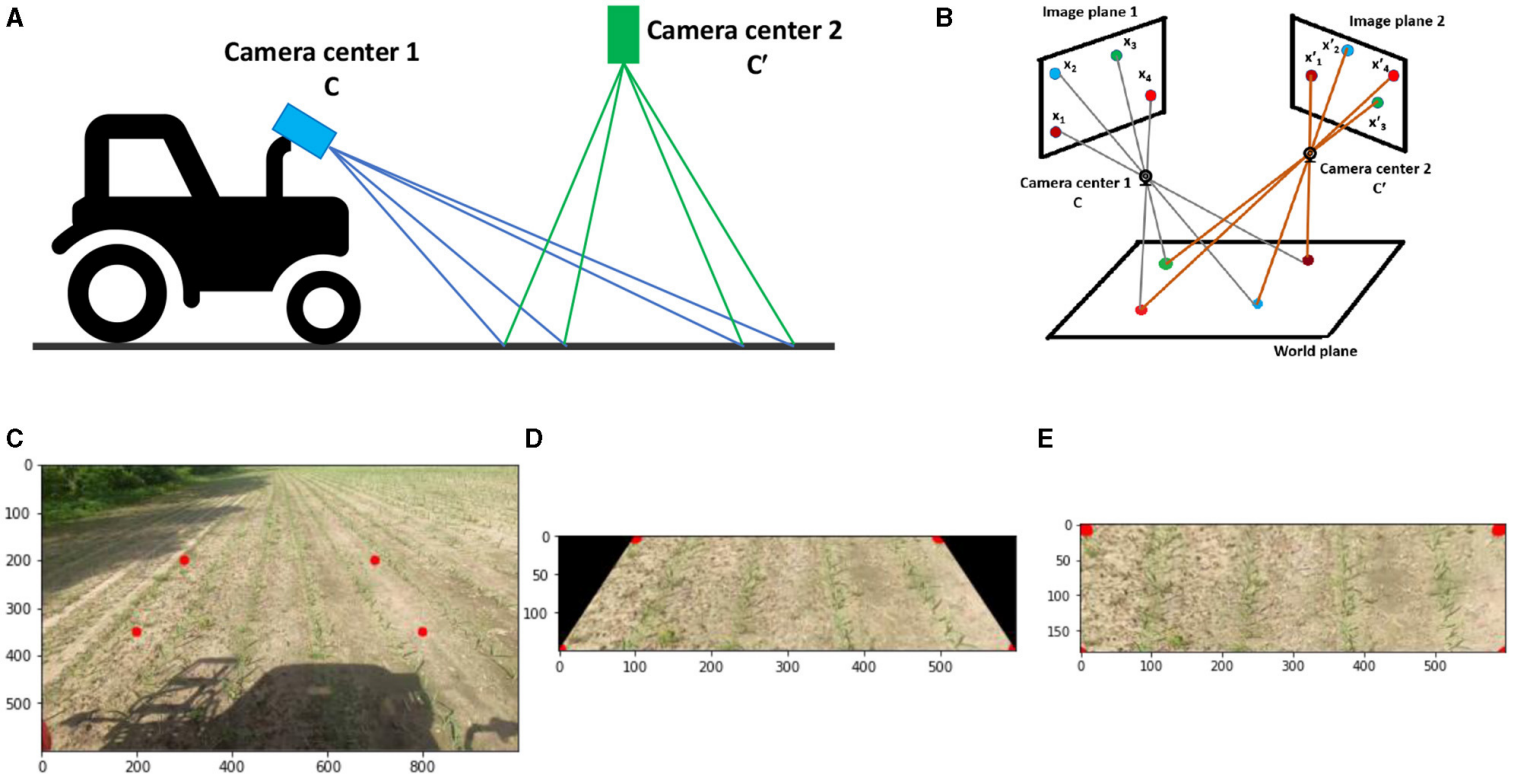
**(A)** Projective transformation employed in the context of Agricultural robotics to convert the perspective of camera 1 view into the virtual perspective of camera 2 view. **(B)** In the case of two camera planes, the relationship between image planes is described by projective transformation, provided that all world points lie on the same plane. **(C)** The Original image captured from camera 1, displays boundary points. **(D)** Cropped image utilizing boundary points. **(E)** Transformed cropped image from camera 1 view to camera 2 view.

Or in short, *x*′ = *H*.*x*, where *H* is the homography matrix. This homography matrix establishes a relationship between the spatial coordinates of a point from the source image plane (image plane 1 in Figures 3A, B) to a destination image plane (image plane 2 in Figures 3A, B). ${\left(x_{1}^{\prime }.x_{2}^{\prime },x_{3}^{\prime }\right)}^{T}$ and ${\left(x_{1}.x_{2},x_{3}\right)}^{T}$ are coordinates of a single point on two image planes. There are eight independent ratios in *H* (*h*_33_ is a scaling factor) which implies that a projective transformation has eight degrees of freedom (Hartley and Zisserman, 2003). Here, homogeneous coordinates are used to represent the points.

Let's consider a pair of inhomogeneous matching points (*x, y*) and (*x*′, *y*′) on image plane 1 and 2 respectively. We are considering inhomogeneous coordinates because of their direct measurability from the image plane (coordinates of points in pixels). From Equation 1:


(2) $$\begin{array}{l} x^{\prime }=\frac{x_{1}^{\prime }}{x_{3}^{\prime }}=\frac{h_{11}x+h_{12}y+h_{13}}{h_{31}x+h_{32}y+h_{33}} \end{array}$$

(3) $$\begin{array}{l} y^{\prime }=\frac{x_{2}^{\prime }}{x_{3}^{\prime }}=\frac{h_{21}x+h_{22}y+h_{23}}{h_{31}x+h_{32}y+h_{33}} \end{array}$$

After rearranging:


(4) $$\begin{array}{l} x^{\prime }\left(h_{31}x+h_{32}y+h_{33}\right)=h_{11}x+h_{12}y+h_{13} \end{array}$$

(5) $$\begin{array}{l} y^{\prime }\left(h_{31}x+h_{32}y+h_{33}\right)=h_{21}x+h_{22}y+h_{23} \end{array}$$

where, *x* = *x*_1_/*x*_3_ and *y* = *x*_2_/*x*_3_. Eight linear equations can be formed by using four points on each image plane. It is possible to solve the tomography matrix *H* between two image planes using only four points considering the condition is satisfied that no three points can be colinear (Hartley and Zisserman, 2003). With scale factor *h*_33_ = 1 and four sets of points on each image plane, the following can be formed:


(6) $$\begin{array}{l} \left(\begin{array}{cccccccc} x_{1} & y_{1} & 1 & 0 & 0 & 0 & -x_{1}^{\prime }x_{1} & -x_{1}^{\prime }y_{1}\\ 0 & 0 & 0 & x_{1} & y_{1} & 1 & -y_{1}^{\prime }x_{1} & -y_{1}^{\prime }y_{1}\\ x_{2} & y_{2} & 1 & 0 & 0 & 0 & -x_{2}^{\prime }x_{2} & -x_{2}^{\prime }y_{2}\\ 0 & 0 & 0 & x_{2} & y_{2} & 1 & -y_{2}^{\prime }x_{2} & -y_{2}^{\prime }y_{2}\\ x_{3} & y_{3} & 1 & 0 & 0 & 0 & -x_{3}^{\prime }x_{3} & -x_{3}^{\prime }y_{3}\\ 0 & 0 & 0 & x_{3} & y_{3} & 1 & -y_{3}^{\prime }x_{3} & -y_{3}^{\prime }y_{3}\\ x_{4} & y_{4} & 1 & 0 & 0 & 0 & -x_{4}^{\prime }x_{4} & -x_{4}^{\prime }y_{4}\\ 0 & 0 & 0 & x_{4} & y_{4} & 1 & -y_{4}^{\prime }x_{4} & -y_{4}^{\prime }y_{4} \end{array}\right)\left(\begin{array}{c} h_{11}\\ h_{12}\\ h_{13}\\ h_{21}\\ h_{22}\\ h_{23}\\ h_{21}\\ h_{31}\\ h_{32} \end{array}\right)=\left(\begin{array}{c} x_{1}^{\prime }\\ y_{1}^{\prime }\\ x_{2}^{\prime }\\ y_{2}^{\prime }\\ x_{3}^{\prime }\\ y_{3}^{\prime }\\ x_{4}^{\prime }\\ y_{4}^{\prime } \end{array}\right) \end{array}$$

In order to determine the *H* matrix, boundary points (red dots in Figure 3C) are utilized. The aforementioned points are chosen by us to ensure that the lines formed by the two red dots align parallel to the crop rows. The concept entails that, upon the transformation of the image from image plane 1 to image plane 2 (top view), the crop rows will exhibit parallelism and will not exhibit convergence toward infinity. This will facilitate the he ability to distinguish between crop rows following the clustering process, hence allowing for the imposition of precise straight-line boundary conditions on each fitted line. After the *H* matrix is computed, it is then utilized to perform a transformation on the whole image plane 1 (Figure 3D) resulting in the conversion to the image plane 2 (Figure 3E). From Figure 3E, it is apparent that the crop rows demonstrate parallel alignment rather than converging toward infinity.

##### 3.1.1.2 Segmentation

When provided with an input image in the RGB color space, the system separates each channel as follows: R = red channel, range [0–255] G = green channel, range [0–255] B = blue channel, range [0–255] After the channels have been divided, the subsequent normalization procedure is implemented, which is widely utilized in agronomic image segmentation (Gée et al., 2008):


(7) $$\begin{array}{l} \begin{array}{c} r=\frac{R_{n}}{R_{n}+G_{n}+B_{n}}\\ g=\frac{G_{n}}{R_{n}+G_{n}+B_{n}}\\ b=\frac{B_{n}}{R_{n}+G_{n}+B_{n}} \end{array} \end{array}$$

where *R*_*n*_, *G*_*n*_ and *B*_*n*_ are the normalized RGB coordinates ranging from 0 to 1 and are obtained as follows:


(8) $$\begin{array}{l} R_{n}=R/R_{max},G_{n}=G/G_{max},B_{n}=B/B_{max} \end{array}$$

Here, *R*_*max*_, *G*_*max*_, *andB*_*max*_ represent the maximum values of R, G and B channels, respectively. To prevent division by zero, a minute constant is added to the denominator during the normalization process. The extraction of green color (representing vegetation) can be achieved by the utilization of the following equation (Ribeiro et al., 2005):


(9) $$\begin{array}{l} Excess\;Green,ExG=2g-r-b \end{array}$$

##### 3.1.1.3 Noise reduction

The application of morphological opening and closing operations on binary images is commonly employed to reduce noise. Opening and closing operations are classical image processing operations where opening removes small object (white pixels) from the binary image and closing removes small holes (black pixels in a white pixel blob) from the binary image (Dougherty, 1992).

#### 3.1.2 Step 2: cluster

An effective clustering technique for crop row detection must exhibit two key characteristics: the ability to differentiate between distinct crop row clusters within the region of interest (ROI) without prior knowledge, and robust, intuitive tuning parameters that allow the algorithm to adapt to a wide range of scenarios. Our proposed method utilizes HDBSCAN (McInnes et al., 2017) as the clustering technique.

The algorithm has been designed with preventive measures to reduce incorrect clustering. If there are fewer than three clusters within the region of interest (ROI), the algorithm assumes that some of the clusters have merged due to a high amount of weed (see Figure 1, step 2.2). It then iteratively deletes outliers (weeds) to find crop row clusters. In certain instances, the presence of excessive weed growth or intermittent crop growth may result in the generation of several clusters using HDBSCAN for a single crop row. The manipulation of the *min cluster size* parameter enables control over this aspect. For the fixed *min cluster size* parameter, *crop row distance* is used as a threshold parameter (Figure 1 step 2.3). If multiple clusters are in close proximity to one another, (determined by the distance of crop rows), then the cluster with smaller data points and smaller height will be deleted. In Figure 1, the green-colored cluster (step 2.3) will be deleted.

#### 3.1.3 Step 3: line fitting

Random Sample Consensus (RANSAC) (Fischler and Bolles, 1981) algorithm is employed for line fitting on individual clusters owing to its straightforward implementation and robustness. Since RANSAC iteratively determines the most suitable data points for line fitting inside a cluster, it excludes the outliers (possibly weed data points) and shows robustness when weeds are present.

The algorithm for detecting crop rows is fully presented in Algorithm 1. The next section discusses important tuning parameters of the algorithm and their effects on crop row detection.

**Algorithm 1:** CAROLIF.


INPUT: color image
OUTPUT: fitted lines over crop rows
Function *ProjectiveTransformation* (color image, ROI, boundary points):
Use ROI to crop the input image
Use boundary points for projective transformation
*return* transformed image
Function *SegmentAndClean* (transformed image):
Binary segment image using ExG and Otsu
Noise reduction with morphological operations
*return* binary segmented clean image
Function *ClusterImage* (segmented image):
Cluster segmented image with HDBSCAN
Check if correct number of clusters present
If not, delete outliers and check again
*return* crop row clusters
Check if crop row cluster's distances are correct
Delete incorrect clusters
Fit straight line on crop row clusters with RANSAC
Check if fitted lines are within slope threshold
Plot straight lines over crop rows and show

### 3.2 Pseudocode (CAROLIF)

The *ProjectiveTransformation* function accepts an RGB image of full size, together with inputs specifying the size of the region of interest (ROI) and the border points. The input image is cropped utilizing “ROI size.” Boundary points refer to the four corners of the cropped image that serve as reference points for its transformation. This function returns the projective transformed cropped image where crop rows are parallel to each other. The *SegmentAndClean* section of the algorithm use the cropped projective modified image as its input. Initially, the binary picture is generated from the color image using *ExG* and Otsu thresholding techniques, wherein green pixels are assigned a value of 255, while all other pixels are set to zero. Subsequently, morphological opening and closing processes are employed to effectively eliminate noise artifacts from the image. The *ClusterImage* phase of the algorithm accepts the clean segmented image as its input. The use of HDBSCAN to the segmented image enables the identification and delineation of distinct clusters. Occasionally, the interconnection of numerous crop rows might occur as a result of elevated weed pressure or atypical crop development. The observed clusters exhibit a significantly larger quantity of data points in comparison to conventional crop row clusters. In the event that this particular situation occurs, a process of iterated outliers deletion is initiated and continues until the connected crop rows become separated. The function returns the number of clusters, along with the location of each cluster's data point, that are considered to represent crop rows. Subsequently, two assessments are conducted utilizing geometric expertise pertaining to the arrangement of crop rows. In the three-dimensional world, it is typical for crop rows to maintain a consistent spatial separation. The distance is converted into a pixel value on the camera image plane, and subsequent evaluations are conducted to determine if two clusters are located within this specified distance. If the answer is yes, it can be inferred that one of the entities under consideration is a cluster of weeds. The identification and removal of weed clusters is determined by assessing their height and the quantity of data points. Once the process of fitting straight lines through each cluster is accomplished, the final check is conducted. Given that projective transformation is being employed, it is expected that each fitted line will exhibit a slope in close proximity to 90°. The angular orientation of the lines changes from 90° due to the turning of the agricultural vehicles. In this investigation, a slope threshold range of [70, 110] degrees is utilized. The lines that fall outside of this range of slopes are excluded from the final result. Ultimately, the plotted lines are superimposed into the identified crop rows.

### 3.3 Performance comparison

In this study, we compare the proposed method to four other methods. The first one, Hough transform (Hough, 1962), is a feature extraction technique in digital image processing. Following the initial stage, the Canny edge detection technique (Canny, 1986) is employed to extract the edges from the binary image. Through the process of coordinate transformation, the colinear points located along the edges of the binary image are changed into concurrent lines inside the parameter space by the use of a voting mechanism. The Hough transform is a method used to detect lines by accumulating votes. The primary tuning parameter in this context is referred to as the “threshold,” which represents the minimal number of intersections required to identify a line. However, it should be noted that this particular parameter lacks intuitiveness. Moreover, a minor modification of the parameter significantly alters the resulting outcome. In addition, the Hough transform often produces a significant number of false positive lines in the absence of any filtering mechanism. In this study, we have applied a filtering process to exclude lines with low slopes and lines that are in close proximity. However, the process of choosing the appropriate threshold values for the slope and determining which lines are considered adjacent lacks robustness. The output exhibits significant variations depending on these characteristics.

The second method is referred to as the Sliding-window method. After step 1 (Figure 1), a window with dimensions of 20 by 20 slides over the ROI. During this process, the algorithm determines the center coordinates of the white pixel clusters contained within the window. By traversing the whole ROI, the center points of the crop rows are derived. Next, a linear regression model is used to fit a least-squares straight line to the center points of the crop rows. Some variants of this method are available in literature (Garćıa-Santillán et al., 2017; Zhang et al., 2018). One of the primary constraints associated with this approach is the determination of the precise location of the crop row initiation point, as well as the identification of the distinct segments within which each row is situated. Consequently, the presence of a significant weed population greatly diminishes the precision of this approach.

The third method is Template Matching followed by Global Energy Minimization (TMGEM) (Vidović et al., 2016). It uses dynamic programming in order to achieve efficient global energy minimization. This methodology is capable of functioning effectively without the need for any pre-existing knowledge regarding the number of crop rows. Furthermore, it exhibits a reasonable level of insensitivity toward weed presence and is compatible with various stages of crop growth.

The fourth method is named Cluster-Least squares. After step 2 (Figure 1), the least squares straight line fitting is used. The least squares method operates by minimizing the sum of squared errors to achieve the smallest attainable value. Consequently, this approach exhibits sensitivity toward outliers. The presence of outliers, specifically in the form of weed, introduces bias in the least squares method, hence diminishing the accuracy of crop row detection.

The final method is named CAROLIF and is the proposed method. Other than TMGEM, all the other methods are built and implemented from scratch by us.

### 3.4 Performance metric

**Figure 4:**
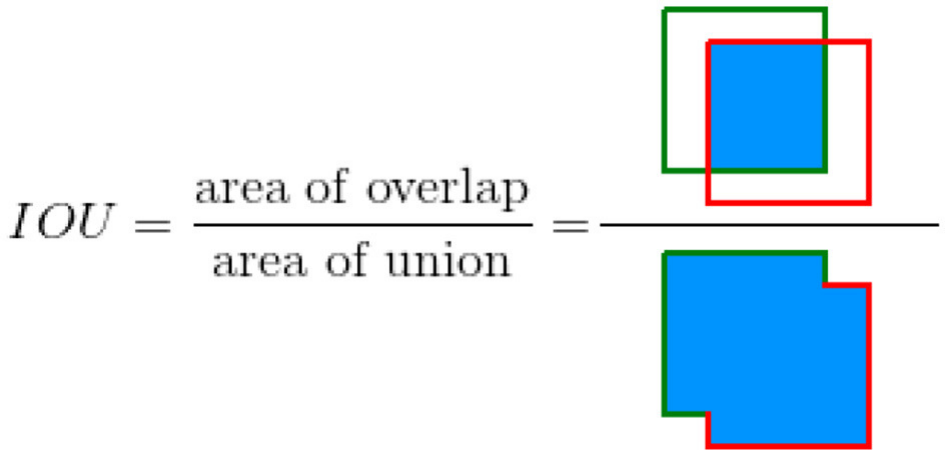
IOU between a ground truth bounding box (in green) and a detected bounding box from algorithm (in red).

**Figure 5:**
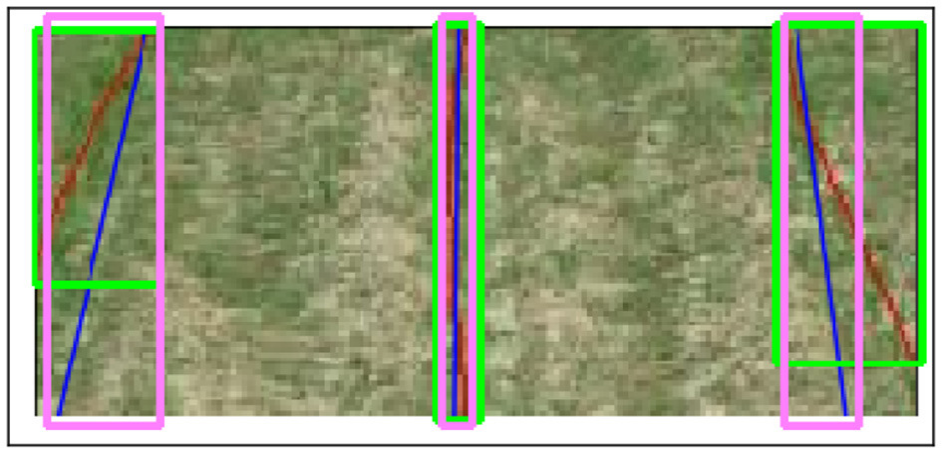
Actual bounding boxes. The red lines represent the ground truth detection of crop rows, while the blue lines represent the algorithm's detection of crop rows. The green bounding boxes help to encapsulate the height and width of the ground truth lines. The pink bounding boxes capture the height and width measurements of algorithm detection lines.

The accuracy of each method is quantitatively measured using the intersect over union (IOU) metric. The evaluation of the overlap between two bounding boxes is performed by IOU. In order to perform the task, both a ground truth bounding box and a forecasted bounding box are necessary. Subsequently, the algorithm computes the proportion of the shared area between two bounding boxes in relation to the overall combined area of the bounding boxes. Figure 4 (Padilla et al., 2020) shows the visual representation of IOU. Figure 5 illustrates the process of generating bounding boxes for the specific scenario of crop row detection. IOU is an intuitive parameter. A score of 1.0 signifies that the predicted bounding box corresponds precisely with the actual bounding box. A score of 0.0 indicates a complete absence of overlap between the predicted bounding box and the true bounding box.

To evaluate the accuracy and recall of the detections, we counted the number of true positive and false positive crop row detections. The overall accuracy was calculated based on these true positive and false positive values. For recall, we considered the number of false positive, false negative, and true positive detections to provide a comprehensive assessment of the algorithm's ability to correctly identify crop rows while minimizing errors.

## 4 Results

### 4.1 Qualitative comparison

**Figure 6:**
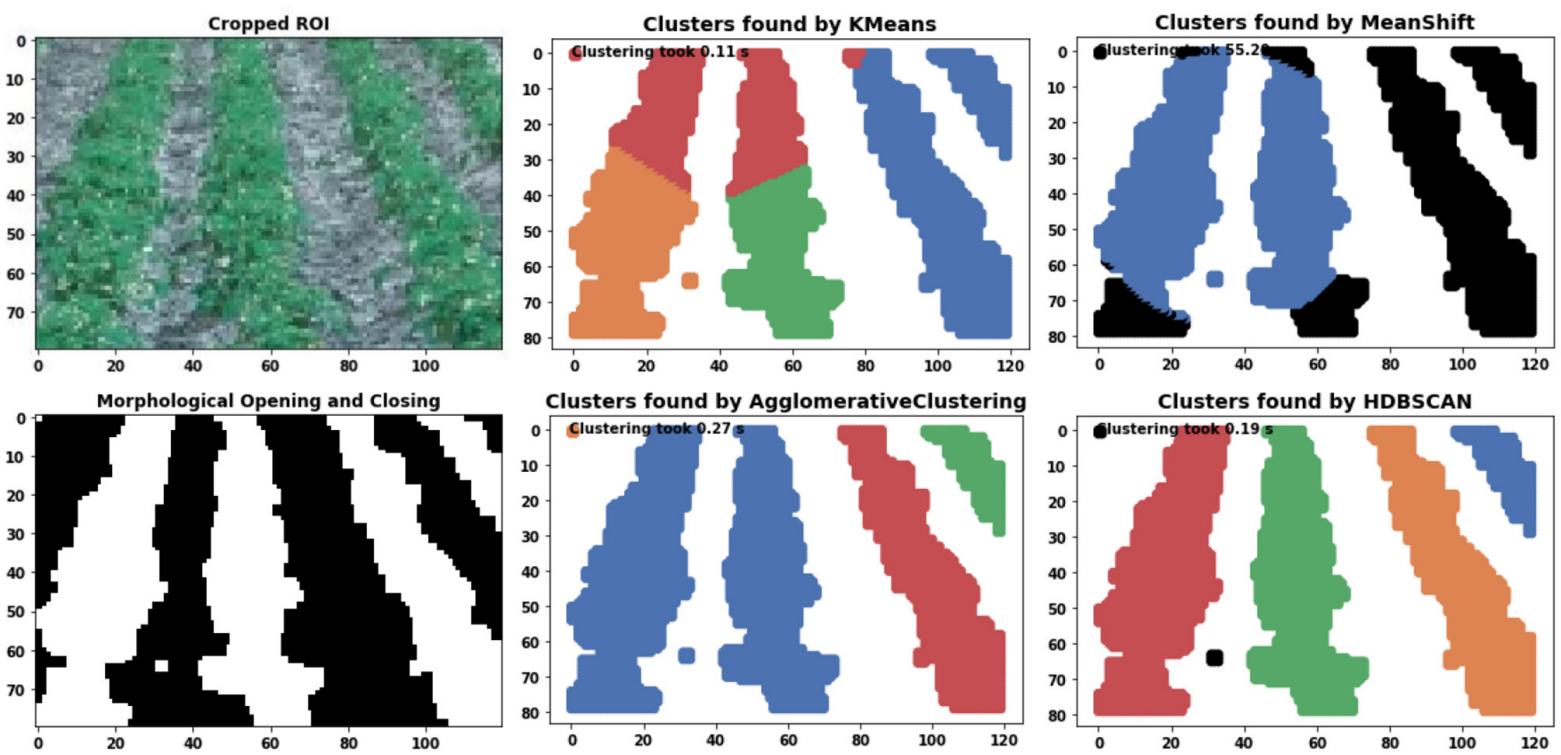
Comparison of Kmeans, MeanShift, Agglomerative and HDBSCAN clustering algorithm on crop row detection. The runtime is displayed in the top left corner of each image. Distinct colors are used to represent separate clusters. The presence of black colors is indicative of outliers.

To understand the proposed method's clustering capability of crop rows, we conducted a comparative analysis of four different clustering techniques. Figure 6 shows the performance output of Kmeans (Arthur and Vassilvitskii, 2006), Meanshift (Comaniciu and Meer, 2002), Agglomerative (Pedregosa et al., 2011), and HDBSCAN (McInnes et al., 2017) on crop row detection. Kmeans (Arthur and Vassilvitskii, 2006) fails because of the anisotropic nature of the crop row data which means they are elongated along a specific axis. The Kmeans algorithm, because of its equal treatment of all data points, does not effectively differentiate any local variation within a cluster. The advantage of Meanshift over Kmeans is that we don't have to specify the number of clusters. The Meanshift algorithm is predicated on the assumption of an underlying probability density function for the given data and it proceeds by locating centroids at the maxima of the density function. The default parameter *bandwidth* which dictates the size of the region to search through, shows wrong results. Additionally, this approach exhibits significantly slower performance compared to alternative methods that have been examined, rendering it unsuitable for real-time applications. The agglomerative clustering technique is a hierarchical approach that groups data into clusters based on their similarity. The clustering process starts by initially considering each individual data point as a distinct cluster. Subsequently, the clusters are iteratively merged together until a predetermined criterion is satisfied. However, in order for this approach to be effective, it is necessary to have prior knowledge of the “number of clusters.” HDBSCAN is a density-based method that extends DBSCAN (Ester et al., 1996) into a hierarchical clustering algorithm. The only tuning parameter used is *min cluster size* and its effect is explained in the subsequent sections. From Figure 6 it is clear that HDBSCAN successfully clustered the crop rows. It also identifies the outliers (black pixels) which are then omitted from output clusters.

**Figure 7:**
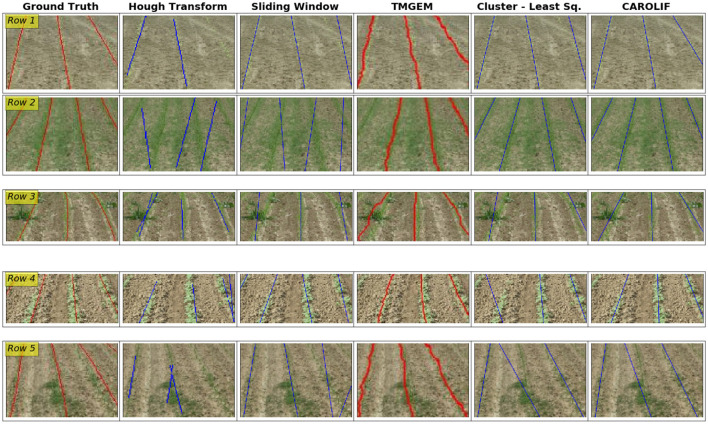
Performance comparison of crop row detection in challenging scenarios for Hough transform, Sliding window, TMGEM, Cluster-Least square and CAROLIF (proposed) methods with ground truth results. (Row 1) intermittent, very early crop growth, no weed. (Row 2) early crop growth stage, significant presence of weed, resulting in the interconnection of crop rows. In this case, crop rows and weeds are not differentiable. (Row 3) early crop growth stage, unusually large intermittent weeds, causing the crop rows to take on curved shapes. (Row 4) moderate level of crop growth, crop absent from certain rows, no weed. (Row 5) early crop growth stage—intermittent, concentrated weed growth.

In Figure 7 (Row 1), it is difficult to discern the initial phases of crop growth and the arrangement of crop rows with the naked eye. The Hough transform and sliding window approaches exhibit a failure in accurately detecting the third row inside the given image. The TMGEM and the proposed approach exhibit superior performance. In (Row 2), the connection between crop rows 2 and 3 can be attributed to the presence of high weed pressure and the early growth stage of the crops. The difficulty in resolving this issue arises from the high concentration of weeds, which poses a challenge for any line fitting algorithm since it tends to fit a line across the pixels representing the weeds. The Hough transform and sliding-window approach are both methods that align with this pattern. The CAROLIF model demonstrates the most favorable outcome in this particular situation. In (Row 3), there is an occurrence of weeds that exhibit abnormally large dimensions. Most conventional row detection algorithms are ineffective in accurately detecting rows and tend to erroneously fit lines across weed pixels due to the abnormally high concentration of weeds. This is the sequence of events that occurred for all the other techniques. The CAROLIF algorithm addresses this limitation by utilizing the RANSAC method, which iteratively identifies two points with a 99% likelihood of being inliers within a cluster and then fits lines based on these two points. Choi et al. (1997). In (Row 4), the upper portion of the crop row is absent in the initial row. Additionally, in the upper left corner, there are green pixels originating from a neighboring crop row. The clustering algorithm based on least squares is ineffective due to its lack of robustness against outliers. The issue is resolved with the implementation of CAROLIF. The second crop row in (Row 5) CAROLIF exhibits inferior outcomes. This phenomenon occurs due to the significant disparity in growth rates between crop rows and weeds. Consequently, the clustering method eliminates the crop row segment as an outlier. One possible approach to address this issue is to adjust the value of the *min cluster size* parameter in the HDBSCAN algorithm, namely by reducing its magnitude.

### 4.2 Quantitative comparison

**Figure 8:**
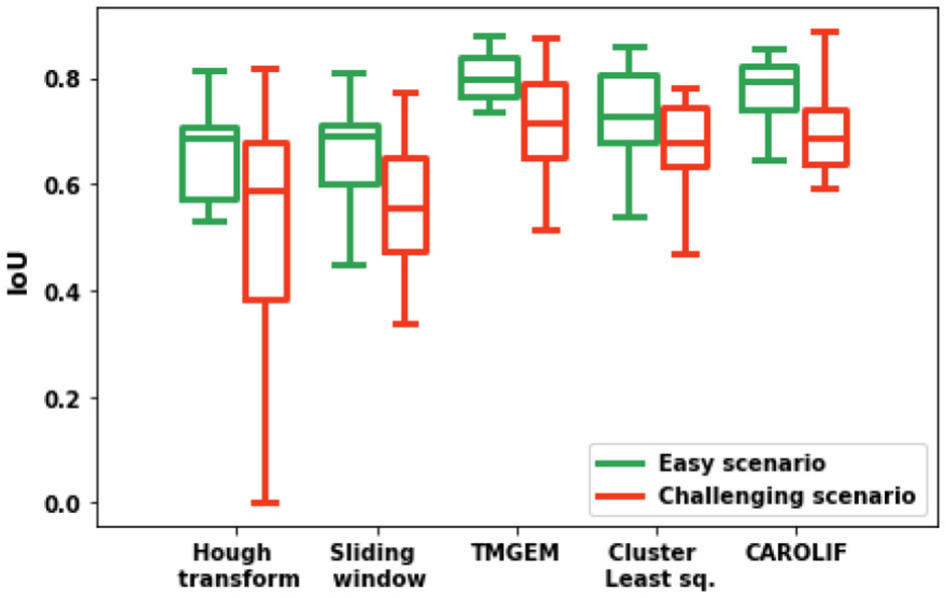
Box plot comparison of IOU values for different methods under easy and challenging scenarios.

A total of fifteen examples are investigated for each scenario (easy and challenging). In each instance, the IOU value is determined by selecting only three rows, ensuring accurate comparison with TMGEM outcomes. (the authors of TMGEM had used three detected lines). Figure 8 shows the performance comparison of different algorithms under easy and challenging scenarios. In regard to all algorithms, it is seen that the IOU value is greater for easy scenarios as opposed to challenging scenarios. This finding suggests that the accuracy of all algorithms is negatively affected by the presence of weeds. The greatest median Intersection over Union (IOU) values for both easy and tough scenarios are observed in TMGEM, with CAROLIF closely following suit. In simplified settings, the TMGEM algorithm exhibits the minimal spread of IOU, indicating its superior consistency in accurately detecting crop rows. In situations that present difficulties, CAROLIF exhibits the narrowest spread and the highest minimum IOU value, indicating that it outperforms other algorithms in harsh conditions with greater consistency. In general, the performance of the Hough transform and sliding window techniques is notably inferior when compared to the other three algorithms. The algorithms TMGEM, cluster-least squares, and CAROLIF exhibit similar performance, with TMGEM demonstrating a little superiority over the other two methods. The mean Intersection over Union (IOU) value for each scenario and the average IOU for all algorithms are presented in Table 3.

**Table 3:** IOU value for different algorithms.

**Algorithm**	**Mean IOU (easy)**	**Mean IOU (challenging)**	**Avg. IOU**
Hough transform	0.64	0.53	0.58
Sliding window	0.66	0.55	0.61
Cluster-least sq.	0.72	0.67	0.7
CAROLIF	0.76	0.69	0.73
TMGEM	0.79	0.71	0.75

**Figure 9:**
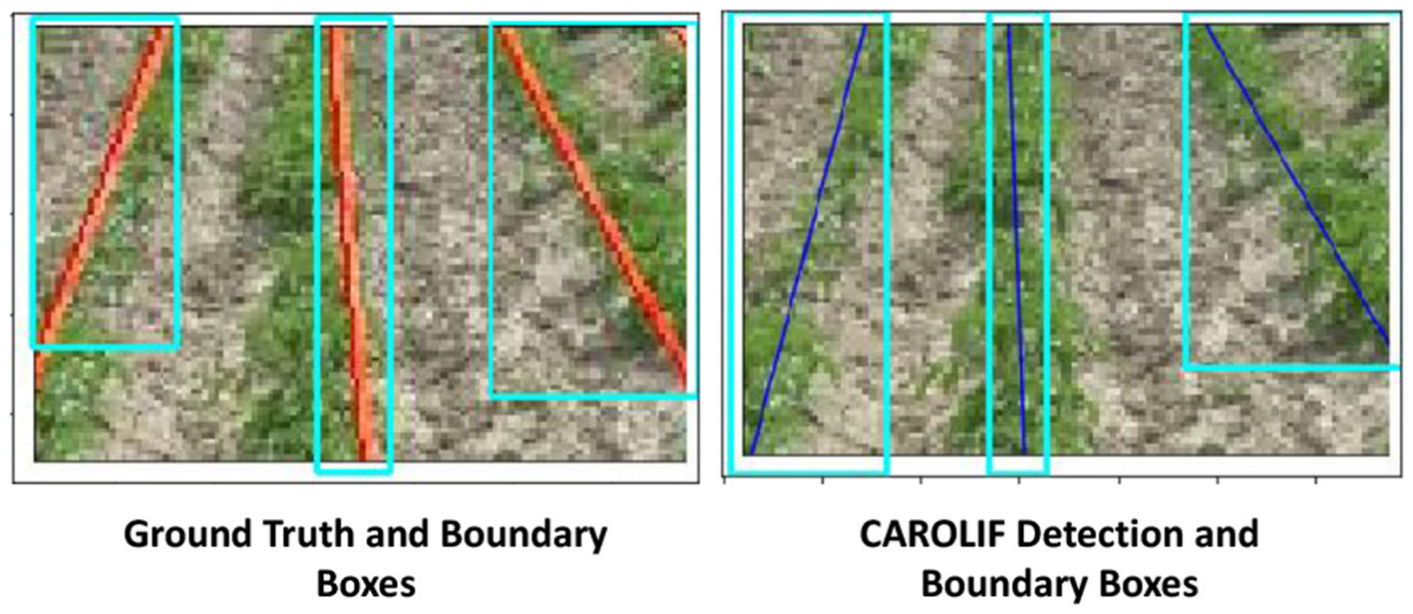
**(Left side)** Ground truth value and boundary boxes from CRBD dataset. **(Right side)** CAROLIF detection and boundary boxes.

The relatively lower performance of the CAROLIF technique, as well as other color-based segmentation methods, might be attributed to the ground truth values of the CRBD dataset. An illustration of a specific example is depicted in Figure 9. The detected ground truth lines (red lines) for the left and center rows are not precisely centered. The ground truth value of the left row exhibits a bias toward the left, while the ground truth value of the center row demonstrates a bias toward the right. A segmentation method based on color is employed to separate the pixels with a green hue from the pixels representing the background. Subsequently, the identified green pixels are utilized to generate the best-fit lines, which are then plotted as rows. Consequently, the regression lines that provide the best-fit pass through the central points of the identified crop rows. In cases where the ground truth value is not located at the center of the crop rows, the IOU value may be reduced, despite the algorithm producing the best-fit line. In Figure 9, it can be argued that the identified lines by CAROLIF exhibit higher accuracy compared to the ground truth value for the left and center rows. The deviation of ground truth values of the CRBD dataset can be one of the main reasons behind the overall inferior IOU value. In the context of comparing color-based segmentation algorithms, it may be stated that selecting the algorithm with the highest IOU value would be the preferable decision.

To compensate for the possible bias of CRBD dataset, we also calculate the accuracy of the methods on the CRBD dataset. We count the number of true positive and false positive detection of crop rows. From those true positive and false positive values, we calculate the overall accuracy. IOU shows us, when crop rows are detected, what percentage of detected crop row pixels overlap with ground truth values. With accuracy, we just calculate how many crop rows are detected correctly. Ground truth values from the CRBD dataset are not needed because human operator can judge which crop row is detected correctly. Table 4 shows the accuracy of different algorithms on CRBD dataset. The accuracy of CAROLIF and TMGEM is significantly better than all other algorithms in both scenarios. Moreover, CAROLIF has 100% accuracy in easy scenarios and the highest accuracy in challenging scenarios.

**Table 4:** Accuracy of different algorithms in easy and challenging scenarios.

**Algorithm**	**Accuracy (easy)**	**Accuracy (challenging)**
Hough transform	91.1%	77.7%
Sliding window	84.6%	73.9%
Cluster-least sq.	93.7%	82.2%
CAROLIF	100%	95.7%
TMGEM	100%	95.2%

### 4.3 Processing time

**Figure 10:**
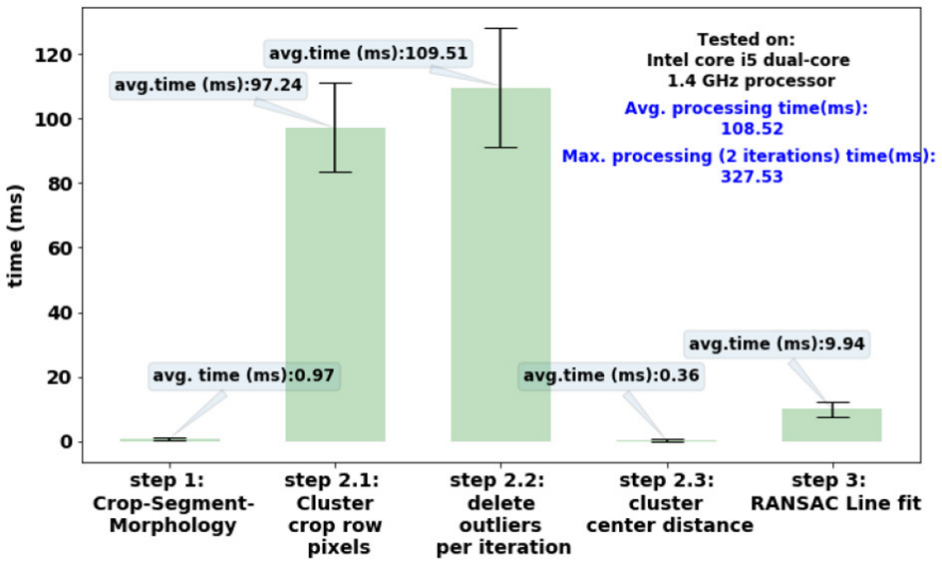
Processing time of each step of CAROLIF crop row detection algorithm. ROI size (120 by 80) pixels.

Figure 10 shows the processing time for each step of CAROLIF crop row detection algorithm. With the development of very powerful embedded hardware like the Jetson TX2 which has built-in video processing capabilities, the proposed algorithm demonstrates the ability to provide real-time performance, even when applied to high-speed vehicles.

Table 5 presents a comparative analysis of processing time. It is important to reiterate that the current implementation does not utilize parallel-processing or multi-threading techniques, which have the potential to dramatically decrease processing time. Clustering algorithms exhibit much longer computational time in comparison to the Hough transform or Sliding window techniques. The TMGEM algorithm requires significantly more time to execute compared to clustering approaches, rendering it impractical for real-time crop row recognition applications.

**Table 5:** Processing time for different algorithms.

**Algorithm**	**Processing time (ms)**	**Avg. FPS**
Hough transform^*^	2.02	495
Sliding window^*^	2.88	347
Cluster-least sq.^*^	100.12	9.9
CAROLIF^*^	108.52	9.2
TMGEM^**^	1750	0.5

^*^Core i5 dual-core 1.4GHz processor. (120 by 80) ROI size.

^**^Core i5 quad-core 3.3GHz processor. (320 by 240) ROI size.

### 4.4 Performance on video input

**Figure 11:**
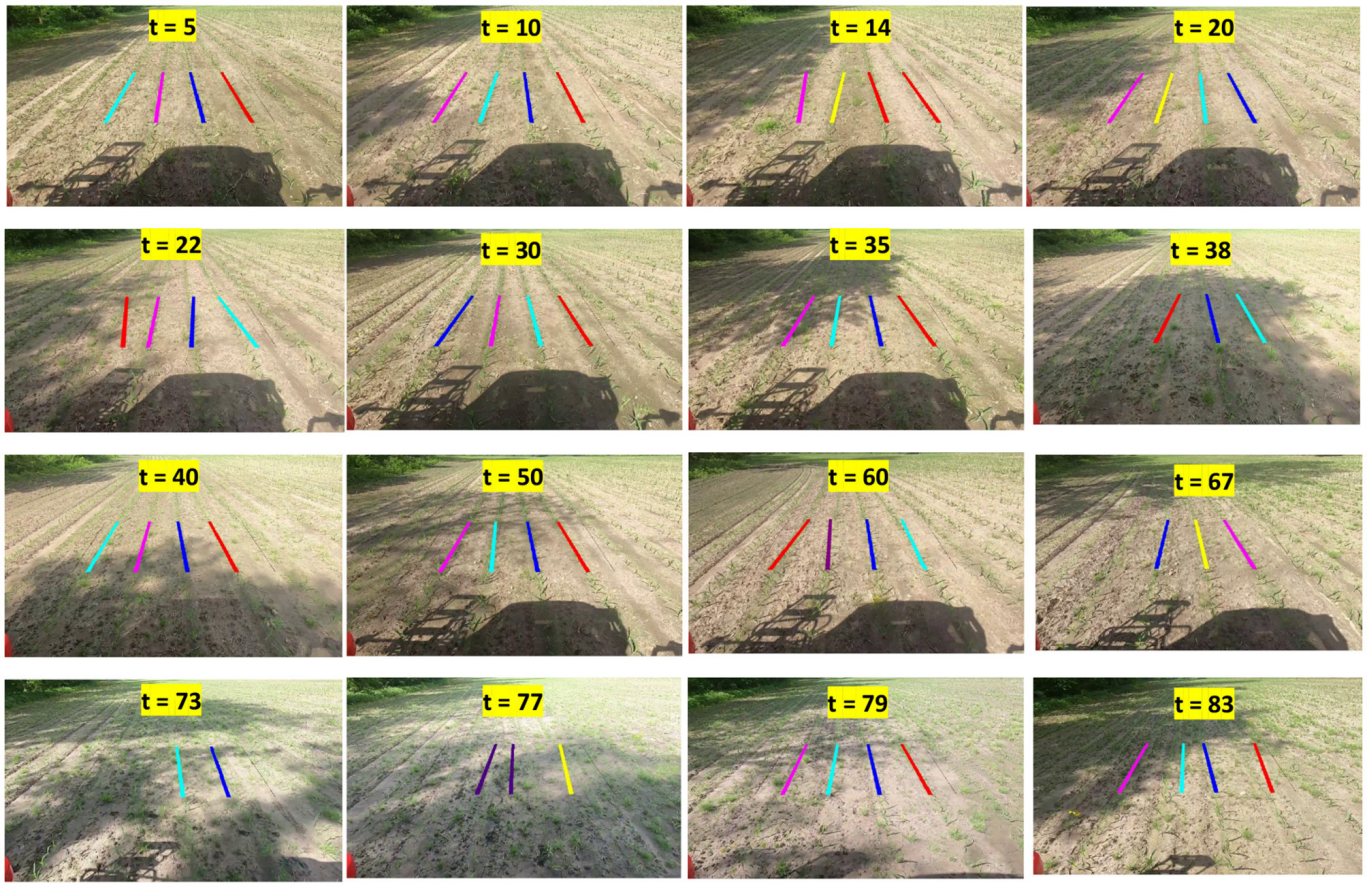
Performance of CAROLIF algorithm on real-time video footage obtained from an agricultural vehicle.

The performance of the CAROLIF algorithm in detecting crop rows on real-time video collected from an agricultural vehicle is seen in Figure 11. The time step *t* = 5 indicates that this frame is extracted from a video at a time of 2.5 s. The notation *t* = 10 indicates that this particular frame is extracted from a movie at the time interval of 5 s. During the time interval from *t* = 5 to *t* = 20, the crop rows are well-lit by the sun, resulting in minimum proliferation of weeds. The CAROLIF system demonstrates robust performance and accurately identifies all crop rows. At time point *t* = 22, there is observable weed growth occurring between the two leftmost rows, accompanied by the presence of shadow. Consequently, the detection of the left row exhibits a certain degree of inaccuracy. However, at time *t* = 30, upon transitioning to the well-illuminated region, accurate detection of all crop rows is achieved. The scenarios characterized by *t* = 35 and *t* = 38 present additional challenges, namely the presence of strong weed growth where the height of the weeds is nearly equivalent to that of the crop, as well as the interference caused by shadows. However, the accuracy of crop row detection is consistent across all scenarios. During the time interval from *t* = 40 to *t* = 60, there is a noticeable presence of sparse shadows and weed growth. However, CAROLIF demonstrates strong performance in these somewhat tough settings, accurately detecting all crop rows. At time point *t* = 67, the tractor undergoes a left turn, causing the left crop row to exceed the slope threshold [(70,100) degrees] and so be excluded from the result. One potential solution to this issue is to adjust the slope threshold. However, the remaining three crop crows are accurately identified. One of the most demanding cases that a crop row detection system can encounter at *t* = 73 and *t* = 77. The presence of shadows and sunlight is observed, accompanied by a significant proliferation of weed growth and a limited and sparse development of crops. In these settings, accurately detecting crop rows is a challenging task even for human visual perception. Consequently, the performance of CAROLIF experiences a decline. However, when reaching a somewhat improved state at time *t* = 79, CAROLIF demonstrates the capability to accurately recognize all four crop rows.

### 4.5 Effectiveness of projective transformation

**Figure 12:**
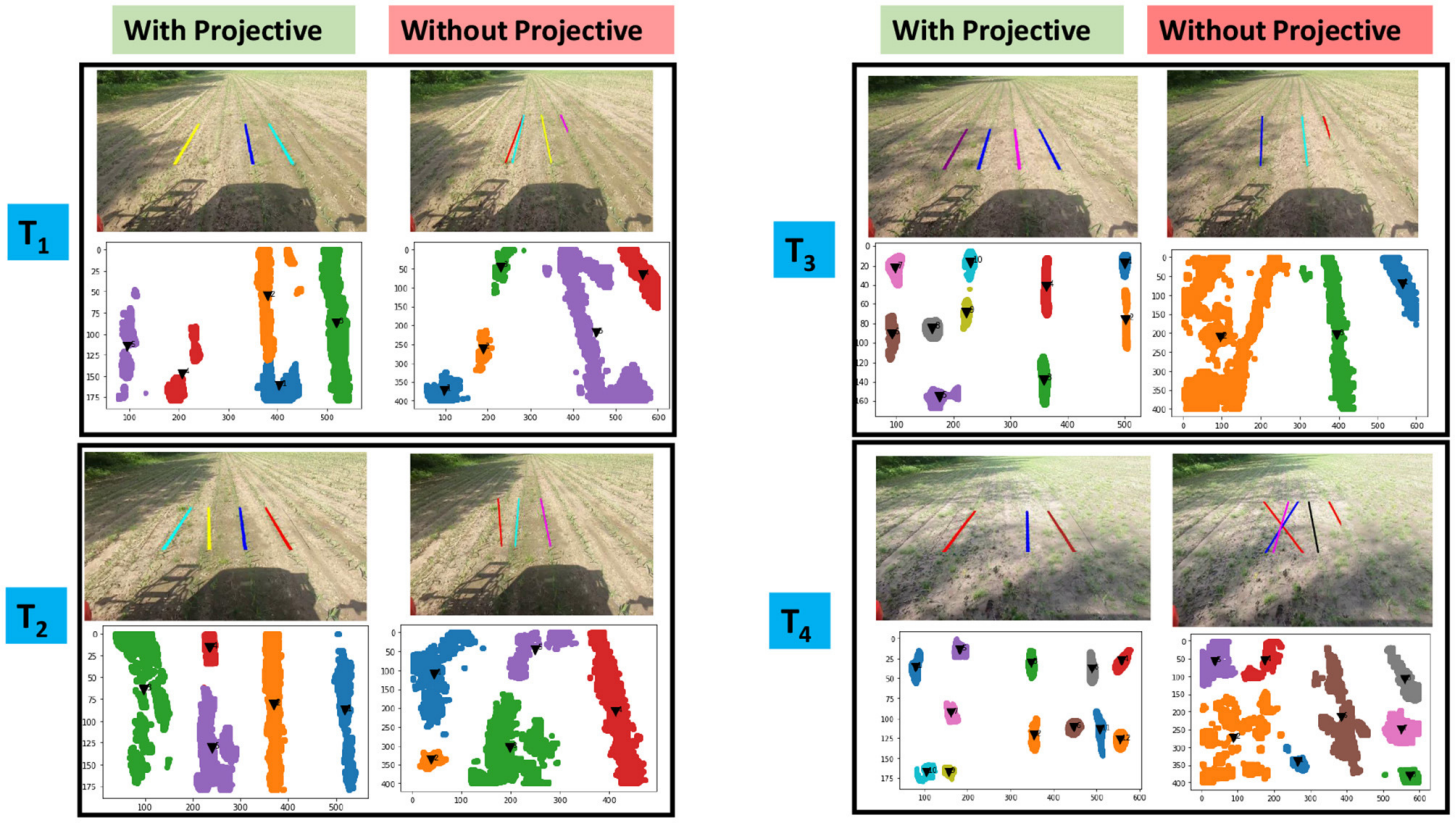
Comparison of crop row detection results with and without projective transformation. Four different scenarios are chosen. (*T*_1_) and (*T*_2_): sunny, no weed, no shadow, intermittent crop growth. (*T*_3_): sunny, no weed, shadow present. (*T*_4_): high weed pressure, shadow, intermittent crop growth.

Figure 12 illustrates the efficacy of projective transformation in mitigating false positive crop row detection in intricate scenarios. Four distinct frames, each corresponding to a different condition, have been chosen for the purpose of comparison. During time periods *T*_1_ and *T*_2_, the prevailing weather conditions are favorable with intermittent crop growth. The utilization of projective transformation results in the parallel alignment of clusters, hence facilitating their differentiation. In contrast, the absence of projective transformation results in the random dispersion of clusters, making it challenging to differentiate them based on crop row distance and a rigid straight line threshold. Consequently, in the absence of projective transformation, the CAROLIF algorithm inaccurately assigns lines to clusters. The cases involving *T*_3_ and *T*_4_ exhibit increased complexity due to the presence of darkness, severe weed pressure, and intermittent crop development. Based on the analysis of the cluster output, it is evident that a significant number of cluster cores remain after the removal of outliers. The application of projective transformation, employing rigorous thresholds, facilitates the elimination of inaccurate cluster cores and enables the fitting of exclusively accurate lines. Particularly at *T*_4_, it presents a challenge for unaided human vision to accurately perceive the alignment of crop rows. However, CAROLIF demonstrates success in detecting three out of four crop rows. Furthermore, the system only displays the lines that are deemed appropriate and omits those that may not be suitable, hence minimizing the occurrence of false positive detection. The performance of CAROLIF is assessed by measuring its effectiveness on both the video with and without projective transformation. In the absence of projective transformation, a number of frames exhibit a lack of significant detection, leading us to exclude these frames from our calculations. The quantification of false positive, false negative, and true positive crop row detections is conducted by video analysis. Subsequently, accuracy, precision, and recall are computed based on these measurements. The information is presented in Table 6. The CAROLIF model demonstrates a high level of accuracy, over 90%, when applied to real-time video analysis involving intricate conditions such as shade presence, intermittent crop growth, and significant weed pressure. The results indicate that the performance of CAROLIF is deemed satisfactory for real-time applications. Additionally, it demonstrates the enhancement of CAROLIF's performance in all aspects through the utilization of projective transformation.

**Table 6:** Performance of CAROLIF on video data.

	**With projective transformation**	**Without projective transformation**
Accuracy	90.5%	77.1%
Precision	96.6%	84.5%
Recall	93.3%	89.8%

The capability of CAROLIF to accurately identify crop rows in real-time, even in fairly demanding settings, is evident. In instances where the human eye alone may struggle to accurately identify crop rows, the precision of detection diminishes. However, the incorporation of distance threshold and slope threshold effectively mitigates the occurrence of false positive detections of crop rows, even in very demanding settings. In the context of real-time applications, there are two potential avenues to enhance performance. Initially, it is possible to rely solely on the GPS signal until the tractor reaches a specific location where accurate detection is achieved for all four rows. Additionally, the implementation of a tracking algorithm might be considered as a potential solution to address the issue of deteriorating sensor output or detection accuracy in highly tough settings. This algorithm would aim to monitor the centers of crop rows, so providing a means to mitigate the impact of these challenging conditions. When included into the autonomous navigation framework of an agricultural robot, the aforementioned fail-safes can be implemented into CAROLIF in order to enhance its robustness.

## 5 Discussion

The proposed CAROLIF algorithm demonstrates robust crop row detection capabilities across a diverse range of scenarios. Our comparative analysis of various clustering methods revealed that HDBSCAN is particularly effective for clustering crop rows, which is a key component of the CAROLIF algorithm. This method's ability to handle varying densities and its robustness against outliers contributed significantly to the reliable performance of CAROLIF. Furthermore, we evaluated the performance of CAROLIF along with four benchmark algorithms: Hough Transform, Sliding Window, TMGEM, and Cluster-Least Square method in different challenging scenarios. CAROLIF exhibits consistent performance in these scenarios, whereas some of the other algorithms fail in some cases. The quantitative comparison further highlighted CAROLIF's strengths, as it exhibited the lowest variation in Intersection over Union (IOU) during testing on the samples of the CRBD dataset. This consistency is a crucial factor for reliable performance in real-time applications. While the average IOU for the Cluster-Least Square, TMGEM, and CAROLIF methods were comparable—0.70, 0.75, and 0.73 respectively—CAROLIF stands out due to its higher overall accuracy and lower image processing time. These attributes make it particularly well-suited for real-time implementation, where speed and precision are critical. Moreover, in tests using video feeds, CAROLIF successfully detected crop rows within its region of interest (ROI) for the majority of time frames. This performance underscores the algorithm's reliability in dynamic, real-world conditions. The incorporation of projective transformation in CAROLIF was instrumental in reducing false positives and improving accuracy, particularly in scenarios with significant perspective distortions. By aligning clusters along the correct axis, projective transformation enhanced the precision and recall of detected rows, thereby increasing the overall robustness of the algorithm.

## 6 Conclusion and future work

This study introduces a new approach for crop row detection that utilizes clustering techniques. The algorithm is subsequently evaluated using both static and video datasets. The method under consideration utilizes a clustering technique and incorporates prior knowledge regarding the geometric arrangement of crop rows in order to distinguish between weeds and crop rows. By implementing a smaller region of interest (ROI), the processing time is reduced and enables more precise fitting of straight lines to moderately curved crop rows. In addition, the method incorporates the utilization of RANSAC, a robust line fitting technique, in order to enhance the mitigation of weed interference on the detection of crop rows. The algorithm under consideration demonstrates suitability for real-time implementation on low-specification hardware, namely those lacking a GPU. Furthermore, it exhibits commendable accuracy in detecting crop rows, even in extremely challenging situations. When comparing the performance of other algorithms, CAROLIF demonstrates superior results (IOU 0.73) in comparison to the Hough transform (IOU 0.58), sliding window (IOU 0.61), and cluster-least sq. (IOU 0.7). Furthermore, in both easy and challenging scenarios, CAROLIF demonstrates a high level of efficacy in accurately detecting all crop rows. Conversely, the other approaches exhibit a complete failure in certain scenarios. Accuracy data of different algorithms on CRBD dataset shows that CAROLIF has 100% accuracy in easy scenarios and the highest accuracy in challenging scenarios. Good accuracy on diverse scenarios and low computation time (108 ms) make CAROLIF a good choice for real-time application. The examination conducted on video data obtained from an agricultural vehicle has yielded encouraging outcomes, indicating its potential for real-time implementation. In our study, we were able to attain an accuracy rate of 90.5%, precision rate of 96.6%, and recall rate of 93.3% when analyzing a video that presented intricate scenarios involving shadow, intermittent crop development, and heavy weed pressure. The ultimate objective of this study is to deploy the algorithm on a commercially available single-board computer (Jetson TX2) in order to achieve real-time detection of crop rows from video input.

The proposed algorithm was validated through testing in a cornfield across various scenarios. For future work, it can be evaluated on different types of crop fields with varying row spacing and weed types to assess its generalization capability. Additionally, testing the algorithm under diverse weather conditions, such as cloudy, rainy, and windy environments, would provide insight into its performance under different lighting and dynamic conditions. Further research could focus on fine-tuning the algorithm's parameters and exploring techniques to reduce computation time.

## Data Availability

The raw data supporting the conclusions of this article will be made available by the authors, without undue reservation.
